# Integrating MIKE model simulations with CNNs for rapid and accurate urban flood prediction

**DOI:** 10.1016/j.isci.2026.116187

**Published:** 2026-06-01

**Authors:** Jian Chen, Yangyang Tian, Luyao Zhang, Haizhou Wang

**Affiliations:** 1 School of Water Conservancy, North China University of Water Resources and Electric Power, Zhengzhou 450046, China

**Keywords:** Civil engineering, Computer modeling, Hydrology, Urban planning

## Abstract

Rapid prediction of urban pluvial flooding is an important tool for mitigating current urban flooding disasters. This paper constructs a fast prediction model for urban flooding based on a machine learning approach. Firstly, MIKE numerical model simulations with high accuracy results are used as the data driver, and then the convolutional neural network (CNN) urban pluvial flooding model is structured based on CNN principles. An empirical study was conducted in the urban area of Zhoukou City to validate the proposed model. Results show that the proposed 1D-CNN model achieves a mean prediction error of 5.74% for inundation depth at the waterlogging-prone locations, and completes inference for a 3 h rainfall scenario in approximately 8 s, demonstrating both high accuracy and near-real-time computational efficiency for emergency urban pluvial flooding prediction. Therefore, the CNN urban pluvial flooding model trained by learning can quickly predict results with high accuracy and can support emergency response.

## Highlights

• Physics-based MIKE simulations are used to train a 1D-CNN flood surrogate
• The proposed framework supports rapid prediction of urban pluvial flooding
• The model reproduces inundation dynamics at typical waterlogging-prone locations
• Local topography and drainage characteristics strongly influence ponding behavior

## Graphical abstract

**Figure:**
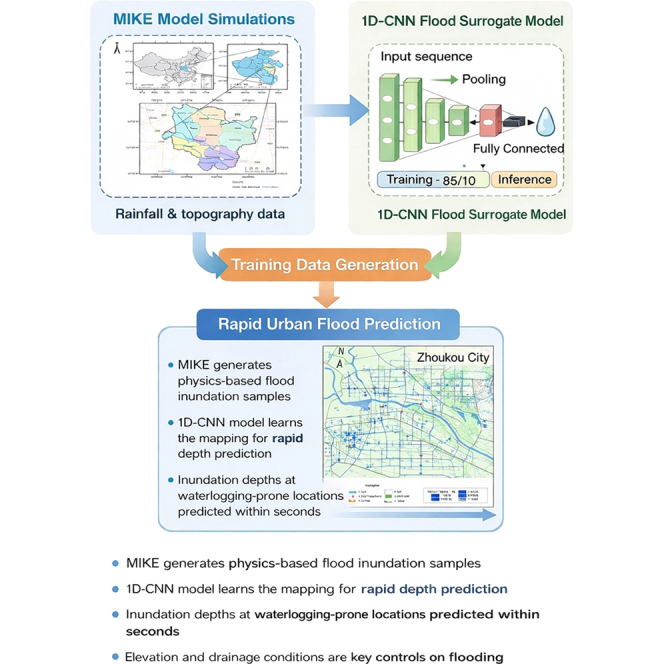

## Introduction

Recent observations indicate an increase in the atmosphere’s moisture-holding capacity, which can intensify extreme precipitation. At the same time, warmer temperatures have increased evaporation from the oceans, accelerating atmospheric water circulation processes and leading to an increased probability of extreme rainfall and extreme drought events.^1^ The combination of urbanization and global warming has led to an increase in the intensity and frequency of urban rainfall, faster runoff generation and concentration/shorter time of concentration, reduced infiltration, and increased surface runoff, making urban flooding an increasingly prominent problem. For example, in 2012, Beijing was hit by the “7–21” rainstorm, which caused an average rainfall of 215 mm in the urban area and up to 460 mm in the center of the storm, affecting 1.9 million people, causing direct economic losses of CNY 11.64 billion and 79 people dead and missing. In 2014, the “3–31” rainstorm in Shenzhen accumulated a maximum of 318 mm of rainfall, causing flooding in more than 200 places, and two people died as a result of the storm flooding.^2^ On July 20, 2021, a 24 h rainfall of 552.5 mm fell in Zhengzhou City, which was paralyzed by that storm, in which the low-lying tunnels represented by Jingguang Road and the underground space represented by metro line 5 were flooded by rainwater, resulting in 292 deaths and 47 missing persons within Zhengzhou City and causing direct economic losses of CNY 53.2 billion.^3^^,^^4^ This shows that heavy rainfall flooding causes huge losses to cities, societies, and people. Therefore, the establishment of an urban storm urban pluvial flooding (urban waterlogging) model for accurate simulation and prediction is an effective means to alleviate the increasingly severe urban flooding, and can provide technical support for urban pre-disaster warning, flood prevention, and mitigation.

Currently, most scholars have conducted a lot of research on numerical hydrological modeling and inundation forecasting. Liu Jun^5^ proposed a mathematical model of rainfall flooding based on the Storm Water Management Model (SWMM) model using rainfall losses to calculate the produced flow, the power wave approximation method to calculate the ground sink flow and the motion wave solution to solve the pipe flow, and the model was simulated and tested in Tianjin city with good results. Michael Gomez et al.^6^ coupled the Hydrology Engineering Center-River Analysis System (HEC-RAS) 1D hydraulics model with the integrated regional hydrological forecasting system (IRF) by integrating meteorological and water level information to improve the forecasting capability of short- and medium-term flood inundation models. Amarnath^7^ et al. based a two-dimensional (2D) surface diffuse flow model, coupled with a hydrological modeling system, to map inundation areas and water depth in rural areas of Sri Lanka and provide preventive measures for urban flooding. Zeng Zhaoyang^8^ et al. coupled a 2D hydrodynamic surface diffuse flow model (LISFLOOD-FP) and an underground pipe network model SWMM, which can accurately simulate the water depth and inundation extent of heavy rainfall in Dongguan city, providing a scientific reference for the prediction and early warning of urban flooding. He Fafa et al.^9^ used geographic information system (GIS) combined with SWMM model to obtain urban flooding simulation results under different recurrence period rainstorm scenarios, and also carried out a study on the risk zoning of flooding in Leopard Creek community in Wuhan to obtain flooding hazard distribution maps for different recurrence period rainstorm scenarios to facilitate the subsequent development of disaster prevention and mitigation tasks. Hou Yan et al.^10^ used the inundation hazard map of the urban area of Kaifeng city as a reference and carried out coupled simulations using the GIS software and the MIKE (DHI’s integrated hydrodynamic modelling system for water flow and flood simulation) model with a rainwater pipe model and a 2D surface diffuse flow model, and the results of the runs can be derived from the ponding process, inundation duration, and water velocity and direction of extreme precipitation in Kaifeng city. Sun et al.^11^ constructed the north canal MIKE 21 model to simulate the dynamic process of flooding under the 50 year event scenario of the wetland construction of the north canal, and analyzed the regular characteristics of flood water flow. These physics-based models can reproduce inundation location and depth with high fidelity; however, they require extensive input data and are computationally expensive, limiting their use for emergency forecasting.

In recent years, with the rapid development of artificial intelligence technology, machine learning technology, with its universality and efficiency, has been widely used and shown outstanding advantages in the fields of atomic nuclear physics, medicine, data mining, and so on.^12^^,^^13^^,^^14^ To compensate for the shortcomings of numerical models, scholars have gradually applied machine learning methods to the prediction of storm water flooding. Liu Yuanyuan et al.^15^ proposed a new method for predicting urban flooding risk based on a combination of back propagation (BP) neural networks and numerical models. The HEM lab team^16^ used deep convolutional neural networks (CNNs) to learn the relationship between the inputs and outputs of a 2D hydrodynamic model, i.e., the relationship between the upstream flow and the water depth at each grid in the computational domain, greatly reducing the computational time required to obtain the flood water depth while maintaining the accuracy of the flood water depth. Nguyen^17^ et al. proposed to apply long- and short-term memory network (LSTM) to quantitative precipitation forecasting (QPF) and combined with urban flooding model to predict water level and inundation in urban catchment areas, and the experimental results showed that the framework has high practicality. Qian^18^ et al. proposed a deep learning-based data-driven flood forecasting model using adversarial neural networks to extract flood dynamics, aiming to improve the computational speed of physically driven 2D urban flood forecasts controlled by the shallow water equation (SWE). Kan Guangyuan et al.^19^ coupled ANN(Artificial Neural Network, a machine learning model for regression and prediction) with K-neighborhood method to improve the poor prediction ability of ANN and other problems. Mei Song et al.^20^ based on support vector machines for flood forecasting, the model has the advantages of strong generalization ability and fast training. Meanwhile, recent studies in climate and air-quality prediction further demonstrate the capability of neural networks (e.g., ANN, CNN, and LSTM/GRU [Gated Recurrent Unit, a type of recurrent neural network designed for sequential data, widely used in time-series prediction such as flood forecasting]) to capture nonlinearity and lag effects in environmental time series; hybrid architectures such as CNN-LSTM often outperform single models at the monthly scale.^21^^,^^22^^,^^23^^,^^24^^,^^25^ For daily air-quality forecasting, wavelet-neural/deep-learning hybrids can better represent non-stationary multi-scale dynamics, and explainable monolayer tools such as SHAP (SHapley Additive exPlanations, a game-theory based method for interpreting machine learning model predictions by quantifying feature contributions) help identify key drivers and enhance interpretability.^26^^,^^27^^,^^28^^,^^29^These studies show that machine learning techniques also have promising applications in urban flood forecasting. Machine learning is a new way of thinking about urban flood forecasting, with guaranteed accuracy and timeliness.

Modeling and forecasting of floods is a complex time series problem. In practical applications, the most concerned indicators are the height of the highest point of the water level and the time to reach the highest point. Therefore, the establishment of flood forecasting models based on process prediction is closely related to actual forecasting. In recent years, some researchers have found that CNN models perform well in the field of time series-based forecasting. This is because the convolutional operations in CNN models are locally connected, i.e., each convolutional kernel processes only a part of the input data, and this local connection can capture the local relationships of the data, which can lead to a better learning of the features of the data. Also, CNN models can effectively extract useful features from noisy time series.

Although hydrological/hydrodynamic models and machine-learning techniques have been widely used to support urban flood simulation and forecasting, physics-based numerical models (e.g., coupled 1D sewer-2D surface models) remain computationally expensive and thus are often unsuitable for rapid, large-scenario forecasting required in emergency response.^30^ Meanwhile, deep-learning approaches for urban pluvial flooding prediction are still comparatively limited, particularly those that explicitly leverage physics-based simulations as training data to build a fast surrogate model.

The objective of this study is to develop a MIKE-simulation-driven deep-learning framework for rapid prediction of urban inundation depth in Zhoukou City. Specifically, we (1) generate a physics-consistent training dataset using a calibrated MIKE URBAN-MIKE 21 model under multiple rainfall scenarios, (2) design and train a 1D-CNN to learn the mapping from rainfall sequences (and associated urban attributes) to inundation depth, and (3) evaluate the proposed surrogate against observed ponding depths at typical waterlogging-prone locations.

The main novelties and contributions are as follows.

(1) A physics-guided surrogate modeling pipeline is proposed, in which MIKE-based 1D-2D simulations are used to systematically create training samples for deep learning, enabling high-fidelity learning while avoiding the need for massive field observations.
(2) A lightweight 1D-CNN architecture is tailored for fast inundation prediction from rainfall sequences, achieving near-real-time computation suitable for emergency forecasting.
(3) The framework provides explainability by quantifying the relative importance of key urban factors (e.g., elevation and land-use/pipe-network characteristics) on inundation depth, supporting practical flood-risk management.

### Materials and methods

#### Overview of the study area

**Figure 1:**
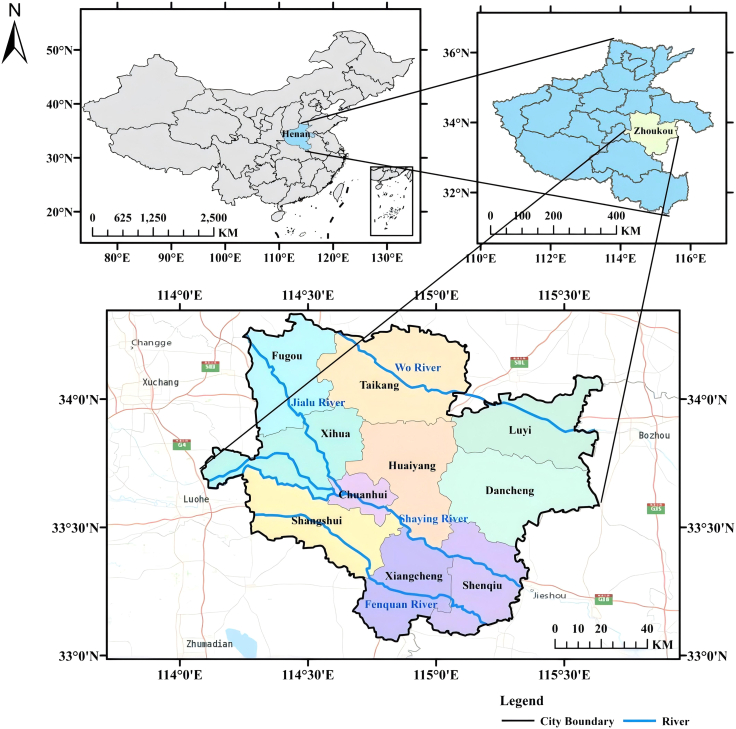
Zhoukou city location map

Zhoukou is located in the East Henan Plain, southeast of Henan Province. The geographical coordinates are 33°03′-34°20′ north latitude and 114°05′-115°39′ east longitude, 135 km wide from north to south and 140 km long from east to west.^31^ See Figure 1 for a map of Zhoukou’s location. Zhoukou City is located at mid-latitude between the Yellow River and the Huai River, alternately influenced by the Mongolian cold high and the Pacific sub-high atmospheric flow in winter and summer, belonging to the warm temperate semi-humid and semi-arid continental monsoon climate. The average annual precipitation in the city is 778 mm, increasing from northwest to southeast. The distribution of precipitation within the year is highly uneven, with a multi-year average of 159 mm in spring from March to May; 413 mm in summer from June to August; 158 mm in autumn from September to November; and 48 mm in winter from December to February. Zhoukou exhibits a short-duration, high-intensity rainfall regime, which tends to generate rapid runoff and high peak flows, imposing strong pressure on the drainage system and increasing waterlogging risk.

Zhoukou City was selected as the case-study area because it represents a typical urban pluvial flooding setting that is frequently affected by short-duration, high-intensity rainstorms, which can rapidly overload the urban drainage system and lead to surface ponding/inundation, thereby imposing strong demands for timely and accurate emergency forecasting. In addition, observed (or surveyed) inundation depths at representative waterlogging-prone locations in the urban area were available for validation, enabling an objective evaluation and comparison of both the calibrated MIKE URBAN-MIKE 21 model and the proposed 1D-CNN surrogate, and thus improving the reliability and practical relevance of the study.

#### Data sources

1 Rainfall data: hourly precipitation data from 2000 to 2021 provided by the Zhoukou Meteorological Bureau. Daily rainfall data from 1971 to 2021 provided by the National Climate Center of the China Meteorological Administration.
2 Pipeline and topographic data: pipeline data for Zhoukou City was provided by the Zhoukou City Natural Resources and Planning Bureau. The topographic data includes elevation point data, sub-bedding type data from Zhoukou city survey and mapping and municipalities and other relevant departments. The spatial resolution of the elevation points is 2 m.^32^

#### Research process

**Figure 2:**
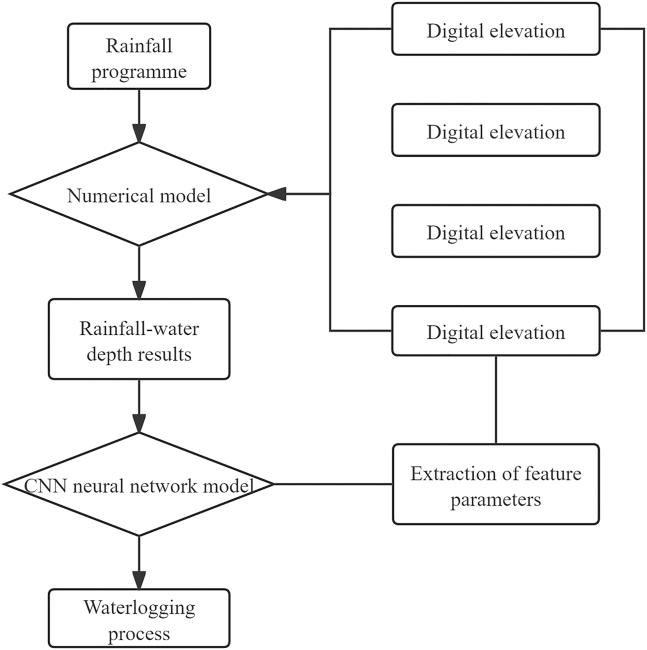
Technical flow chart

In this study, based on the MIKE model, a series of rainfall-water depth data were obtained for different rainfall scenarios as the samples for neural network learning training, and neural network prediction models were built for waterlogging-prone locations respectively, and finally rainfall-inundation samples not involved in the training were used as prediction samples to test the prediction effect of the neural network models. The specific flow chart is shown in Figure 2.

#### Short-duration design storm

The rainfall scenario consists of two components, the short ephemeral design rainfall based on the storm intensity formula and the short ephemeral intense rainfall that is measured to occur.^33^ The design rainfall is based on the storm intensity formula combined with the Chicago design rain pattern over a period of 3 h. The measured rainfall also occurs over a short period of 3 h. The larger the sample database, the more the neural network model learning training and the more accurate the model results. After numerical model simulations, rainfall-flooding samples under various rainfall scenarios are derived. Ninety percent of the samples in the sample database are selected for learning training and 10% for testing.

#### Numerical models

Deep CNNs are data-driven; therefore, paired rainfall-inundation samples are required for training. As it is difficult to collect a large amount of rainfall and inundation data in practical engineering, this paper will use the MIKE inundation model to simulate the collected rainfall data and design rainfall to obtain the water depth data, and use them as training samples to carry out research on the deep CNN model.

The MIKE flood model is based on the MIKE series of software developed by the Danish Hydraulic Institute (DHI), which is powerful, has been tested in many practical projects and is widely recognized in the water research industry. In this paper, a MIKE flooding model will be constructed for the central city of Zhoukou. The model will be parameterized by the measured rainfall and actual water depth collected to confirm the reliability of the MIKE flooding model in the study area. Then, separate inundation simulations are carried out for multiple long-period rainfall events to generate a series of water depth data to provide data support for subsequent machine learning inundation models.

##### Mike urban

MIKE URBAN is a GIS-based modeling software developed to simulate the hydraulic processes of water supply network systems (WD) and drainage network systems (CS).^34^ The core of the calculation is the hydrodynamic model, i.e., the implicit finite difference method is used to solve the one-dimensional (1D) Saint Venant system of equations.^35^ The drainage network system used in this paper consists of two modules, a rainfall runoff model and a pipe flow model, which operate independently of each other. The results of the rainfall runoff model are the volume of runoff generated within each catchment area, which is fed into the pipe flow model as a boundary condition for calculating pipe flow.

The core of the pipe flow model is based on a set of St Venant’s equations for 1D free surface flow, namely the continuum equation (mass conservation) and the momentum equation (momentum conservation—Newton’s second law), which are solved numerically using the Abbott-Ionescu six-point implicit format finite difference method. The calculation method automatically adjusts the time step to the hydraulic process and enables accurate calculation of pipe flows at branch or ring networks. It is also suitable for pressurized, vertically uniform, critical, and supercritical flows, and can accurately simulate flow phenomena such as backflow and overflow.

##### Mike 21

MIKE 21 is a hydraulics calculation software in a 2D plane area that simulates changes in water level and velocity in the horizontal direction.^36^ It is mainly used to simulate water flow, waves, sediment, and environment in rivers, lakes, estuaries, bays, coasts, and oceans. There are two modes of computational kernels commonly used in MIKE 21. One is MIKE 21 for rectangular computational grids, which uses an implicit difference method to solve the 2D Saint Venant system of equations.^37^ The second is MIKE 21 FM for unstructured grids, which uses a cell-centric finite volume method to solve the 2D St. Venant system of equations numerically. The control equations for both are a 2D set of St. Venant equations, that is, a 2D non-constant flow equation consisting of a ontinuous equation and a momentum equation of the following form:

(Equation 2.1) $$\frac{\partial h}{\partial t}+\frac{\partial \left(hu\right)}{\partial x}+\frac{\partial \left(hv\right)}{\partial y}=q\text{,}$$

(Equation 2.2) $$\frac{\partial \left(hu\right)}{\partial t}+\frac{\partial \left(hu^{2}+\frac{1}{2}gh^{2}\right)}{\partial x}+\frac{\partial \left(huv\right)}{\partial y}+gh\frac{\partial Z}{\partial x}+\tau_{bx}=0,\tau_{bx}=g\;\frac{n^{2}u\sqrt{u^{2}+v^{2}}}{h^{1/3}}\text{,}$$

(Equation 2.3) $$\frac{\partial \left(hv\right)}{\partial t}+\frac{\partial \left(huv\right)}{\partial x}+\frac{\partial \left(hv^{2}+\frac{1}{2}gh^{2}\right)}{\partial y}+gh\frac{\partial Z}{\partial y}+\tau_{by}=0,\tau_{\text{by}}=g\;\frac{n^{2}v\sqrt{u^{2}+v^{2}}}{h^{1/3}}\text{,}$$

where “h” represent water depth (m); “Z” represent water level (m); “u,” “v,” “x,” and “y” direction along the vertical average of the horizontal flow component (m/s); “g” is gravitational acceleration (m/s^2^); “n” is roughness; and “q” is the source and sink term (m/s).

Also, “*t*” is time (s); “*x*” and “*y*” are the Cartesian coordinates (m); “*h*” is water depth (m); “*u*” and “*v*” are the depth-averaged velocity components in the “*x*” and “*y*”directions (m·s^−1^); “*g*” is the gravitational acceleration (m·s^−2^); “*z*” is the bed/terrain elevation (m); and “*q*” is the source term (m·s^−1^) representing lateral inflow. The bed shear stress terms “*τbx*” and “*τby*” (m·s^−2^) are computed using Manning’s formulation, where *n* is Manning’s roughness coefficient (s·m^−1/3^).

The study area is a large area, so a structured grid is used to mesh the study area to simplify the work and improve the efficiency of the model calculation, while also providing a good overview of the buildings and roads and other structures in the urban area.

#### Neural network models

##### Principles of deep CNNs

Deep learning, an important branch of machine learning, is essentially the same as machine learning in that it analyses a large amount of sample data and constructs multiple implicit layers to establish feature relationships between inputs and outputs.^38^^,^^39^^,^^40^ Specifically, there are 1D convolutional, 2D convolutional, and three-dimensional convolutional. Among them, 1D-CNNs are suitable for processing sequential data and are also known as time-delay neural networks.^41^ 2D CNNs are the most widely used, and are widely used in computer vision and image processing. Three-dimensional CNNs are generally used in fields such as medicine and video processing. In this study, there is a relationship between the rainfall data and the water depth in the time series, so a 1D CNN was chosen to build the inundation model. The CNN structure used is a 1D convolutional layer (Conv1D), an activation function layer (activation), a normalization layer (batch normalization), a fully linked layer (dense) and a dropout layer.

1 1D convolutional layer

The convolution layer, also known as the feature extraction layer, is set up with multiple filters to convolve the input data, extract its local features, determine the position relationship between the local features, and finally output the feature vector.

2 Activation function layer

The activation function layer, also known as the feature mapping layer, does not change the size of the feature vector but simply maps each element value to another value by means of an activation function, i.e., *x* becomes *f(x)*, *f* being the activation function. The activation function operates by activating a part of the neuron in the neural network and passing the activation information backwards to the next layer of the neural network. Commonly used activation functions include the tanh-function, the sigmoid function and the Relu function, of which the Relu function is currently the most commonly used, with the expression shown in Equation 2.4.

(Equation 2.4) f(x)=max(x,0).

3 Normalization layer

The normalization layer serves to force the feature distribution of the output of the previous network layer into a distribution with a mean of 0 and a variance of 1. At the same time, in order to maintain the feature distribution learned by the previous neural network, i.e., the model expressiveness, two learnable reconstruction parameters γ and β are introduced, whose expressions are shown in Equation 2.5.

(Equation 2.5) $$y=\frac{\left(x-E\left(x\right)\right)}{\sqrt{\text{Var}\left[x\right]}+\varepsilon }\;\cdot \;\gamma +\beta \text{,}$$

where, *y* is the normalized eigenvalue, *x* is the eigenvalue, E(*x*) is the mean of the eigenvalue in the smallest batch, Var(*x*) is the variance, and ε is the standard deviation.

4 Flatten layer

The flatten layer is used to flatten the feature data output from the previous layer, i.e., to make the multi-dimensional feature data 1D, and is generally used as a transition between the convolutional layer and the fully linked layer.

5 Full link layer

The core operation of the full linkage layer is the matrix vector product, which essentially transforms a feature vector from one feature space to another. The purpose of the full linkage layer is therefore to take the previously extracted features and, through non-linear changes, extract the associations between these features and finally map them onto the output space, i.e., to act as a “classifier.”

6 Dropout layer

The dropout layer temporarily removes some neurons during the training of each minimum batch of the neural network with a certain probability until the next training. In the case of stochastic gradient descent, since the removal is random, each minimum batch is training a different network, which helps to reduce the overfitting of the neural network.

##### CNN neural network construction

**Figure 3:**
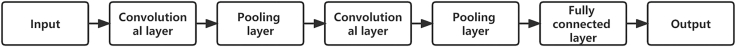
CNN neural network structure diagram

The input and output data are in the form of matrices, and after linear interpolation of the rainfall, the input data are expanded to matrices with a larger number of rows and columns when performing flood-prone location water depth series forecasting. To further extract features from the input data, an additional convolutional layer is considered. At the same time, considering that if the final output size after feature extraction is too large, it will lead to too many features in the output result, a sharp increase in computation and easy overfitting, another pooling layer is added after the additional convolutional layer. The final structure is: input layer-convolutional layer-pooling layer-convolutional layer-pooling layer-fully connected layer-output layer. The structure is illustrated in Figure 3.

##### Model input and output design

**Figure 4:**
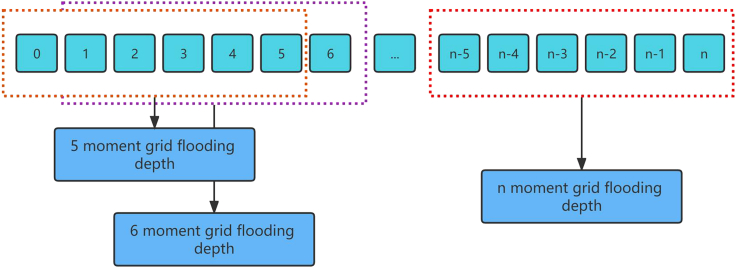
Model input and output design diagram

The input layer of the CNN inundation model receives rainfall data and the output layer is the water depth values for all grid cells at each moment. The water depth at that time is simulated by the rainfall at time *t* and for the five periods before that time, and after each output of the water depth at that time, the input window is rolled back by one time step to simulate the water depth at the next time. The time interval of rainfall data used in this paper is 3 min, i.e., the depth of inundation of all grids in the study area at that moment is simulated by the rainfall process of the first 3 h. When the input is the rainfall process from time 0 to time *n*, the model output is the water depth process for all grids from time 5 to time *n*. The design inputs and outputs are shown in Figure 4.

##### Sample construction and model training

The sample construction process for water depth prediction at urban flooding accumulation points based on CNN neural network models involves the collection, processing, and transformation of data to fit the CNN model. In this paper, we will use the short-duration design storm section count rainfall scenario as Chicago rain type, with general rainfall over the whole area, and the waterlogging depth data are selected to match the results of hydrodynamic model calculations with the corresponding rainfall recurrence period, to derive the sequence of water depth for the corresponding flooding risk control points. Using Python to write a program to batch process the input data, and finally obtain the training set data of five inundation risk control points selected from each drainage area, where the number of training set samples is 432. The neural network model is trained by continuous learning in order to achieve the prediction of the depth of water over time throughout the rainfall process.

The input rainfall sequences were normalized using min-max scaling to the range [0, 0.95], and the inverse transformation was applied to recover the original physical units for evaluation. The dataset was then split into two independent subsets, where 90% of the heavy rainfall-flooding samples were used for training and the remaining 10% were reserved as a held-out testing set. Hyperparameter tuning (grid search combined with 5-fold cross-validation) was conducted within the training set only, and the final performance was reported on the held-out testing set. The optimized hyperparameters (training epochs and learning rate) for the five rainfall sequences are summarized in Table 1.

**Table 1:** Table of model training times and learning rate parameters

Storm waterlogging time series	Number of training sessions	Learning rate
Serial 1	230	0.01
Serial 2	200	0.01
Serial 3	150	0.01
Serial 4	110	0.008
Serial 5	100	0.008

Serial 1–5 correspond to Chicago-type design storms with different return periods, i.e., Serial 1: 10-year, Serial 2: 20-year, Serial 3: 30-year, Serial 4: 50-year, and Serial 5: 100-year rainfall events. The 20-year design rainfall scenario is used for the illustrative analysis in Figure 8.

In this study, multiple rainfall scenarios (Serial 1–5) were considered as model inputs, and all rainfall hyetographs were designed with a consistent duration of 3 h (short-duration design storm). These scenarios were selected to span a representative range of storm magnitudes relevant to urban pluvial flooding based on local rainfall characteristics and common design practice, so that the generated samples capture diverse inundation responses and improve the generalization of the proposed 1D-CNN model for emergency prediction.

**Figure 5:**
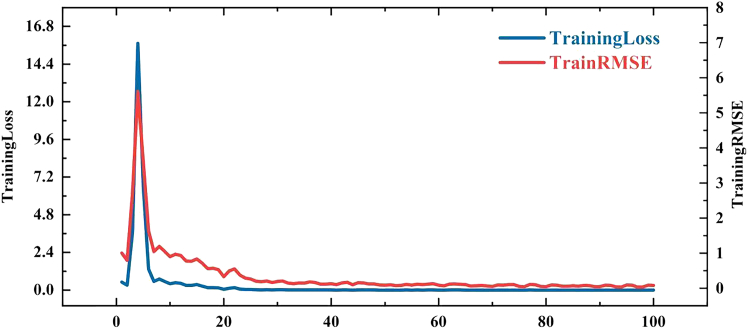
Training loss and RMSE values for sequence 3

Taking the training-prediction process of sequence 3 as an example, the initial learning rate is set to 0.008, and the maximum number of iterations is 100, the specific training process is shown in Figure 5.

As can be seen in Figure 5, the training loss values decrease rapidly within the first 20 steps, indicating a fast learning rate. Both RSME and training loss values reach stability at 80 steps and decrease rapidly to very low levels, indicating that the CNN model is well trained. To examine whether 432 training samples are sufficient, we conducted a sample-size sensitivity analysis by training the 1D-CNN with increasing fractions of the available samples (e.g., 20%, 40%, 60%, 80%, and 100%) and evaluating the performance on the same test set. The results show that the test error decreases rapidly with increasing sample size and then plateaus when the training size approaches the full dataset, indicating that the model performance is near-convergent and that 432 training samples are adequate for this study.

##### Model evaluation methods

To quantitatively evaluate and compare the performance of the proposed model and the benchmark models, six widely used metrics were adopted, including mean squared error (MSE), mean absolute error (MAE), coefficient of determination (*R*^*2*^), percent bias (PBIAS), Nash-Sutcliffe efficiency (NSE), and Kling-Gupta efficiency (KGE). MSE and MAE measure the magnitude of prediction errors, *R*^*2*^ reflects the goodness-of-fit, PBIAS diagnoses systematic bias, while NSE and KGE provide integrated measures of predictive skill that are commonly used in hydrological and flood-model evaluation.^42^^,^^43^^,^^44^^,^^45^^,^^46^^,^^47^^,^^48^

(Equation 2.6) $$\text{MSE}=\frac{1}{m}{\sum_{i=1}^{m}\left({\hat{y}}_{i}\text{−}y_{i}\right)}^{2}\text{,}$$

(Equation 2.7) $$\text{MAE}=\frac{1}{m}\sum_{i=1}^{m}\left|{\hat{y}}_{i}\text{−}y_{i}\right|\text{,}$$

(Equation 2.8) $$R^{2}=1\text{−}\frac{{\sum_{i=1}^{m}\left(y_{i}\text{−}{\hat{y}}_{i}\right)}^{2}}{{\sum_{i=1}^{m}\left(y_{i}\text{−}\bar{y}\right)}^{2}}\text{,}$$

(Equation 2.9) $$\text{PBIAS}=100\times \frac{\sum_{i=1}^{m}\left({\hat{y}}_{i}\text{−}y_{i}\right)}{\sum_{i=1}^{m}y_{i}}\text{,}$$

(Equation 2.10) $$\text{NSE}=1-\frac{{\sum_{i=1}^{m}\left(y_{i}\text{−}{\hat{y}}_{i}\right)}^{2}}{{\sum_{i=1}^{m}\left(y_{i}\text{−}\bar{y}\right)}^{2}}\text{,}$$

(Equation 2.11) $$\text{KGE}=1-\sqrt{{\left(r\text{−}1\right)}^{2}+{\left(\alpha \text{−}1\right)}^{2}+{\left(\beta \text{−}1\right)}^{2}},\alpha =\frac{\sigma_{\hat{y}}}{\sigma_{y}},\beta =\frac{\mu_{\hat{y}}}{\mu_{y}}\text{,}$$

where *m* is the number of samples, ${\hat{y}}_{i}$ and *y*_*i*_ denote the predicted and observed values of sample *i*, respectively, and $\bar{y}$ is the mean of the observed values. In Equation 2.11, *r* is the linear correlation coefficient between $\hat{y}$ and *y*, $\alpha =\frac{\sigma_{\hat{y}}}{\sigma_{y}}$ is the variability ratio, and $\beta =\frac{\mu_{\hat{y}}}{\mu_{y}}$ is the bias ratio, where σ and μ denote the standard deviation and mean value, respectively.

For MSE and MAE, smaller values indicate better performance. For *R*^*2*^, values closer to 1 indicate a better fit. For PBIAS, values close to 0 indicate low systematic bias; positive PBIAS indicates overall overestimation, whereas negative PBIAS indicates overall underestimation. NSE ranges from (−∞, 1], with values closer to 1 indicating higher predictive skill; values below 0 imply the model performs worse than using the observed mean. KGE also attains an optimum value of 1 and jointly reflects correlation, variability, and bias, providing a robust overall assessment of model performance.

## Results and discussion

### Numerical model validation

**Figure 6:**
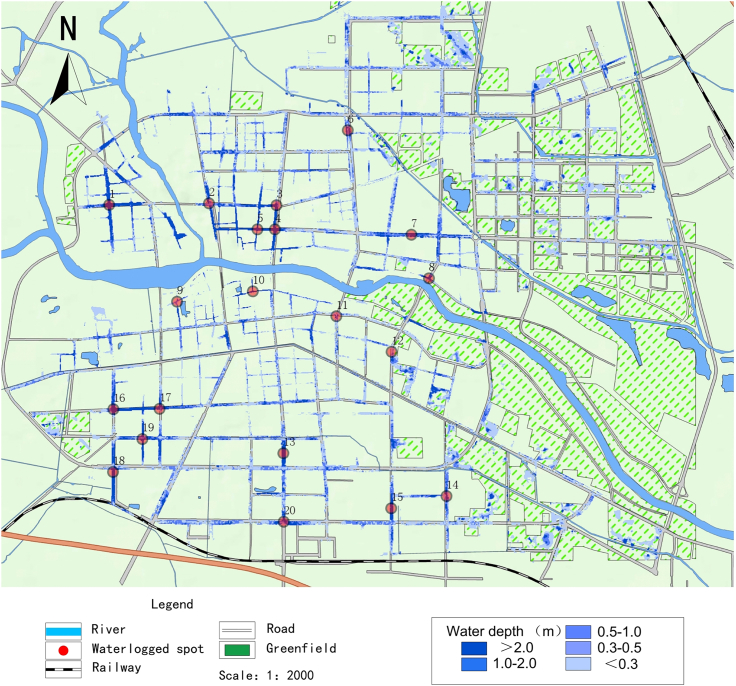
Distribution of major waterlogged sites (2021.7.20)

**Figure 7:**
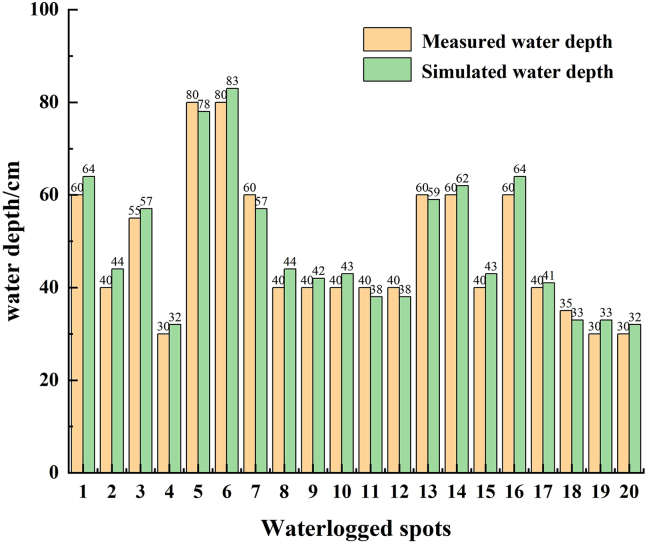
Comparison of measured and numerically simulated ponding depths at inundated water points

Taking Zhoukou City during 2021.7.20 as an example to illustrate, 20 major waterlogging points in the central city were selected and the distribution of waterlogging points is shown in Figures 6 and 7, and Table 3 and show the comparison table and comparison chart between the measured ponding depth and the simulated ponding depth of the numerical model. It can be seen that the error between the measured and simulated values is small, indicating that the numerical model developed is close to reality and qualitatively meets the requirements of neural network learning. The main parameters of the model and their selected values are shown in Table 2.

**Table 2:** Parameter values for the model

Model	Parameter	Value
Mike 11	riverbed roughness	0.025–0.030
Mike 21	dry-wet boundary	dry: 0.002, wet: 0.003
	Eddy viscosity	0.5
	Manning’s number	32
	initial water depth	0
Mike urban	initial rainfall loss	0.5
	decay coefficient	0.9
	pipe roughness	0.015

**Table 3:** Comparison of measured and numerically modeled ponding depths at inundated water points (2021.7.20)

Waterlogged spots	Measured water depth /cm	Simulated water depth /cm	Difference /cm
1	60	65	5
2	40	42	2
3	55	58	3
4	30	31	1
5	80	76	−4
6	80	84	4
7	60	56	6
8	40	45	5
9	40	43	3
10	40	41	1
11	40	38	−2
12	40	42	2
13	60	58	−2
14	60	63	3
15	40	42	2
16	60	63	3
17	40	42	2
18	35	39	4
19	30	34	4
20	30	36	6

### CNN neural network validation evaluation

Table 4 shows the results of the neural network model validation evaluation. From Table 4, it can be seen that the goodness-of-fit R^2^ is above 0.95, indicating that the constructed CNN neural network model is reliable.

**Table 4:** Evaluation of neural validation results

Waterlogged spots	MSE	MAE	*R*^2^	PBIAS (%)
1	0.0020	0.0120	0.9923	6.667
2	0.0050	0.0240	0.9844	10.000
3	0.0090	0.0170	0.9952	3.636
4	0.0030	0.0330	0.9943	6.667
5	0.0040	0.0230	0.9912	−2.500
6	0.0050	0.0110	0.9744	3.750
7	0.0080	0.0220	0.9963	−5.000
8	0.0050	0.0180	0.9866	10.000
9	0.0040	0.0160	0.9784	5.000
10	0.0020	0.0320	0.9964	7.500
11	0.0060	0.0330	0.9866	−5.000
12	0.0080	0.0270	0.9852	−5.000
13	0.0070	0.0210	0.9914	−1.667
14	0.0050	0.0150	0.9945	3.333
15	0.0030	0.0190	0.9922	7.500
16	0.0050	0.0140	0.9955	6.667
17	0.0020	0.0250	0.9956	2.500
18	0.0040	0.0230	0.9944	−5.714
19	0.0070	0.0180	0.9877	10.000
20	0.0060	0.0220	0.9933	6.667
NSE	0.9671			
KGE	0.9685			

PBIAS(%) is computed for each location. NSE and KGE are reported as overall statistics across the 20 locations (NSE = 0.9671, KGE = 0.9685), where values closer to 1 indicate better performance.

### Analysis of the results of CNN neural network for predicting waterlogging

**Figure 8:**
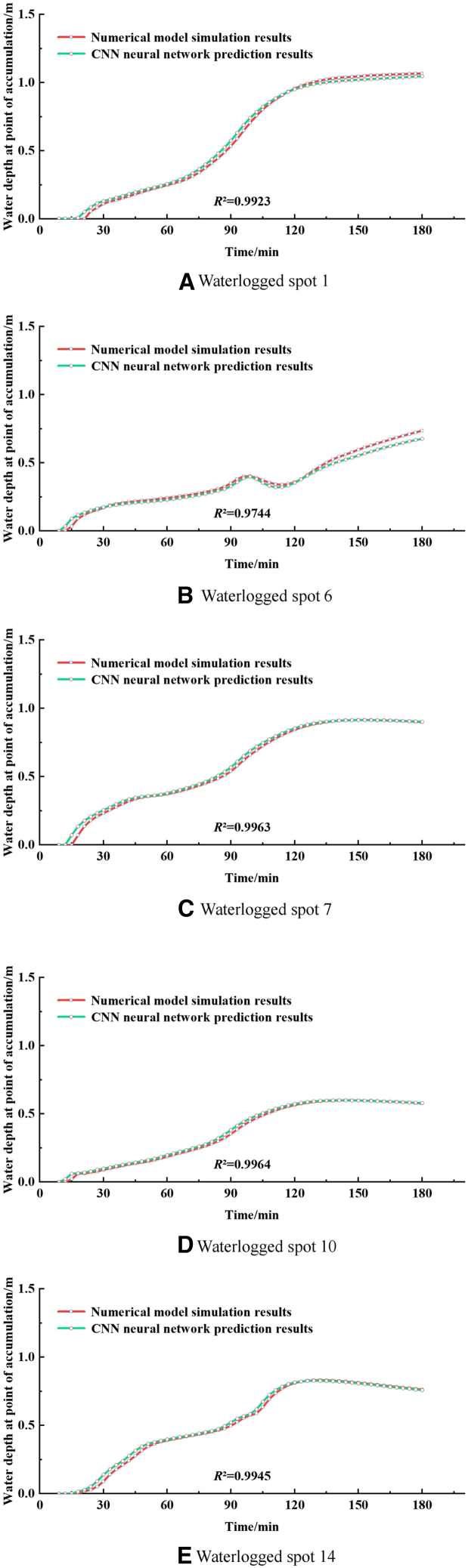
Comparison of numerical model and neural network simulation results

Five of the 20 waterlogged sites were randomly selected for results analysis. Zhoukou City is dominated by single-peak type rainfall with a combined peak ratio of r = 0.5. The storm intensity design formula is based on the Chicago rainfall type, and the actual and design rainfall are predicted separately using the trained CNN neural network model. The Chicago rainfall type with a 20 year design rainfall and a peak rainfall r = 0.5 was selected and the results are shown in Figure 8.

In Figure 8, the prediction accuracy varies with the model mainly because the two models represent the rainfall-ponding process in different ways. The MIKE hydrodynamic model explicitly resolves the 1D sewer-2D surface flow interactions, while the CNN acts as a data-driven surrogate learned from MIKE-generated samples; therefore, the CNN predictions tend to follow the MIKE-simulated hydrographs closely at the selected locations. For the five representative waterlogged spots, the goodness-of-fit remains consistently high (all above 0.97), indicating that the CNN can reproduce both the overall trend and the temporal dynamics of ponding depth produced by the numerical model. In particular, under the Chicago design storm with a peak ratio r = 0.5, rainfall intensity reaches its maximum around 90 min, and both models capture the corresponding rapid rise in ponding depth near this time. Minor discrepancies between the curves (e.g., around the rising limb and near the peak) are mainly attributable to local heterogeneity and numerical sensitivities in the hydrodynamic simulation (e.g., micro-topography and drainage conveyance), whereas the CNN smooths part of these local fluctuations because it learns an average mapping from the sample pool. Overall, Figure 8 demonstrates that the CNN surrogate maintains high accuracy relative to the numerical model across different locations while providing a much faster inference capability.

**Figure 9:**
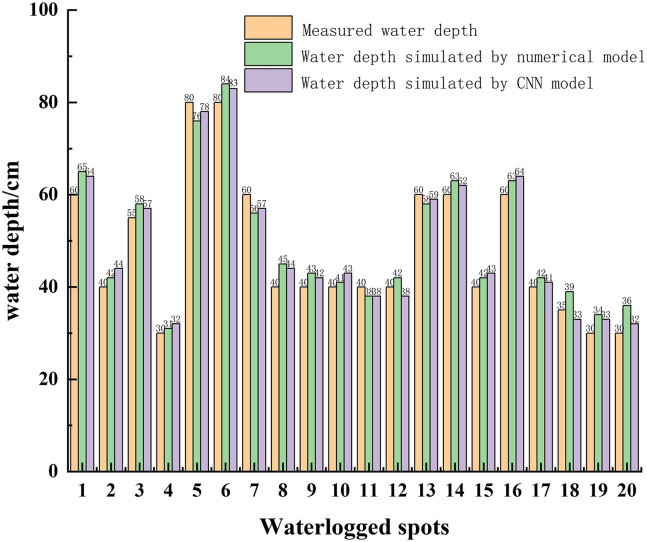
Comparison of measured and modeled water depths at the inundation point (2021.7.20)

Due to the better continuity of design rainfall based on the Chicago rainfall pattern, the continuity of measured data are poor. In order to better reflect the accuracy of the model, predictions were made for the main water accumulation points during 2021.7.20 that did not participate in the training, and the comparison between the predicted results and the measured water depth is shown in Figure 9.

For the 20 major waterlogged sites during 2021.7.20, the average error between the CNN neural network model predicted water depth and the measured water depth was 5.74%. The average error between the numerical model calculated water depth and the measured water depth was 6.97%. It can be seen that the trained CNN neural network model has a high accuracy.

However, in terms of computational speed, neural network models are very fast. The computation time of a numerical model depends on the number of grids, the density of the grids, the extent of the terrain, the configuration of the computer and other conditions. A well-trained neural network model is not subject to these factors. For this simulation, the numerical model takes 10 h to simulate 3 h of rainfall, while the trained CNN neural network model takes only 8 s to calculate the same duration of rainfall. This represents an orders-of-magnitude speed-up relative to the MIKE simulation, providing additional lead time for emergency decision-making. As a result, the neural network model can quickly predict highly accurate results and is able to meet the needs of emergency situations. Therefore, the trained CNN neural network model has advantages in terms of accuracy and computational timeliness for rainfall designed for short ephemeral periods based on the storm intensity formula.

Figure 9 shows that both the numerical model and the CNN reproduce the overall spatial pattern of ponding depth across the 20 waterlogging-prone locations, while noticeable deviations remain at several sites. The numerical model exhibits larger absolute errors at some locations (e.g., spots 20 and 18), where the simulated depths exceed the observations, suggesting potential sensitivity to local drainage conveyance and boundary/tailwater conditions. The CNN predictions are generally closer to the measurements but small overestimation/underestimation still occurs at a few sites (e.g., slight underestimation at spot 18 and overestimation at spots 2 and 16), indicating that local heterogeneity (micro-topography, inlet efficiency, and sewer surcharge conditions) may not be fully captured by the available training samples. Overall, the comparison implies that the CNN surrogate can effectively learn the rainfall-ponding response from the MIKE-generated sample pool and improves accuracy at most locations; however, the remaining discrepancies highlight the need for richer observational data and refined representation of local drainage controls to further reduce site-specific bias.

### Significance analysis of characteristic factors

**Figure 10:**
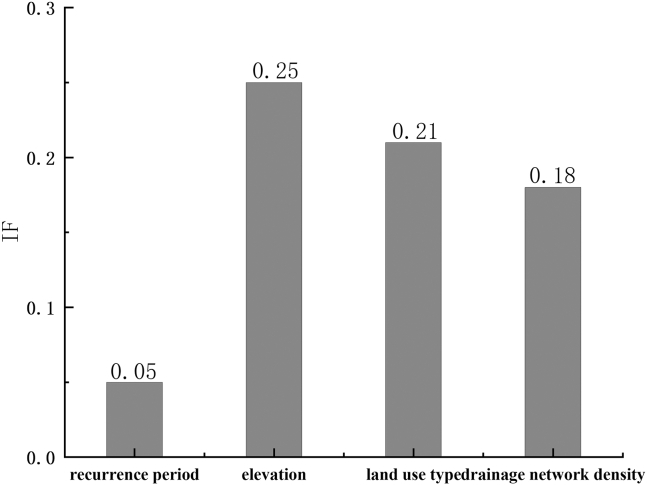
Importance map of characteristic factors

The feature importance (IF) values quantify the relative contribution of each input factor to the CNN-based inundation-depth prediction, where a larger IF indicates a stronger influence on the model output. As shown in Figure 10, elevation has the highest importance (IF = 0.25), implying that micro-topographic controls dominate the spatial redistribution and local accumulation of overland flow, and thus strongly regulate ponding depth. The land-use type (IF = 0.21) ranks second, reflecting the combined effects of surface roughness and imperviousness on runoff generation and routing. The drainage network density (IF = 0.18) also plays a substantial role, indicating that local conveyance capacity and sewer-surface interactions (e.g., surcharge and inlet exchange) are key controls on waterlogging severity. In contrast, the rainfall return period shows a comparatively smaller importance (IF = 0.05) within the designed 3 h scenario setting, suggesting that, once a storm exceeds the drainage threshold, spatial heterogeneity in terrain and drainage conditions becomes the primary determinant of where and how deep ponding occurs. For practical interpretation, features with IF ≥ 0.20 (elevation and land use) can be considered dominant controls, providing guidance for prioritizing terrain-sensitive low-lying areas and land-surface management measures, while drainage network enhancement remains an important engineering lever. Further increasing or changing the dimensions and data volume of the model to study the effect of different combinations of characteristic factors on the model accuracy is an important direction for subsequent research.

### Analysis of the causes of urban pluvial flooding

The main causes of waterlogging at the 20 waterlogging-prone locations are associated with a combination of topographic controls and drainage-system limitations. Many sites are situated in locally low-lying terrain; during intense rainfall, surface runoff is rapidly concentrated along micro-topographic gradients from higher surrounding areas toward these depressions, leading to runoff convergence and ponding. In addition, the storm water conveyance capacity is constrained at some locations due to small pipe diameters and limited hydraulic capacity. When the main outfall is partially blocked, or when the outfall is subject to high tailwater levels in the receiving river (backwater effect), the sewer system can become surcharged and transition to pressurized flow. Under such conditions, manholes and inlets may overflow, causing surface inundation. Waterlogging can be further exacerbated by insufficient pumping capacity at pumping stations, which limits drainage under high-water-level conditions and prolongs the recession of ponded water.

### Limitations of the study

The surrogate model is trained on a sample pool generated by the numerical model, and thus its performance depends on the calibration quality and scenario coverage of the underlying MIKE simulations; moreover, given the limited availability of observed urban waterlogging data, deviations may still occur under extreme or out-of-distribution rainfall conditions. Future work should incorporate richer observations, broaden scenario design, and consider dynamic boundary information to improve generalization and robustness.

Future work will focus on improving the operational applicability of the proposed MIKE-simulation-driven 1D-CNN framework. This includes incorporating more realistic storm scenarios (e.g., spatially non-uniform and moving rainfall) together with uncertainty analysis to enhance robustness under extreme events, integrating dynamic boundary forcing and real-time observations (river stage, pump/gate operation, and monitoring data) for online updating, and extending the current point-based inundation-depth prediction to rapid, higher-resolution inundation mapping while preserving the near-real-time computational advantage for emergency warning and response.

## Resource availability

### Lead contact

Further information and requests for resources and data should be directed to and will be fulfilled by the lead contact, Haizhou Wang (wanghaizhou@ncwu.edu.cn).

### Materials availability

This study did not generate any new unique reagents.

### Data and code availability

• The detailed data associated with the article is available from the lead contact on reasonable request.
• This article does not report original code.
• Any additional information required to reanalyze the data reported in this article is available from the lead contact upon request.

## Acknowledgments

This work was supported by the 10.13039/501100001809National Natural Science Foundation of China (grant no. U22A20237).

## Author contributions

All authors contributed to the study conception and design; writing and editing, J.C. and Y.T.; chart editing, L.Z. and H.W. All authors read and approved the final manuscript.

## Declaration of interests

The authors declare no competing interests.

## STAR★Methods

### Key resources table

**Table:** 

REAGENT or RESOURCE	SOURCE	IDENTIFIER
Hourly precipitation records (2000–2021)	Zhoukou Meteorological Bureau	N/A
Daily rainfall records (1971–2021)	National Climate Center, China Meteorological Administration	N/A
Urban drainage-network pipeline dataset	Zhoukou City Natural Resources and Planning Bureau	N/A
Topographic elevation-point dataset (2 m resolution)	Local survey, mapping, and municipal departments	N/A
Observed/surveyed inundation-depth data for typical waterlogging-prone locations	Field survey and study-area validation dataset	N/A

**Software and algorithms**		

MIKE URBAN	Version: MIKE by DHI 2014	N/A
MIKE 21	Version: MIKE by DHI 2014	N/A
Python	Version: 3.12	https://www.python.org/downloads/release/python-31210/
Deep-learning framework used for the 1D-CNN model	This study	N/A
Chicago hyetograph method	Design-storm construction method used in this study	N/A
Original model-training and inference code	This study	N/A

### Method details

#### Study area

Zhoukou City, located in the East Henan Plain in southeastern Henan Province, China, was selected as the study area. The city experiences a warm temperate semi-humid to semi-arid continental monsoon climate and is characterized by short-duration, high-intensity rainfall, rapid runoff concentration, and frequent urban pluvial flooding. The case area was chosen because representative waterlogging-prone locations with observed or surveyed inundation depths were available for model validation, allowing an objective comparison between the calibrated MIKE URBAN–MIKE 21 model and the proposed 1D-CNN surrogate model.

#### Data sources

The study used rainfall, drainage-network, terrain, and inundation-depth information. Hourly precipitation records for 2000–2021 were obtained from the Zhoukou Meteorological Bureau, and daily rainfall records for 1971–2021 were obtained from the National Climate Center of the China Meteorological Administration. Pipeline data for the urban drainage network were provided by the Zhoukou City Natural Resources and Planning Bureau. Topographic data, including elevation-point data and related underlying-surface information, were obtained from local survey, mapping, and municipal departments, with a spatial resolution of 2 m. Observed or surveyed inundation depths at typical waterlogging-prone locations in the urban area were used to validate the numerical model and the surrogate model.

#### Rainfall scenario design

Rainfall scenarios were constructed using both measured short-duration storm events and design storms. The design storms were generated from the local storm-intensity formula combined with the Chicago hyetograph method, and the rainfall duration was fixed at 3 h. Multiple design-storm scenarios were created to represent different return periods relevant to urban pluvial flooding in Zhoukou City. In the current manuscript, five designed rainfall sequences (Serial 1–5) are reported, corresponding to 10-year, 20-year, 30-year, 50-year, and 100-year rainfall events, respectively. These designed hyetographs were combined with the MIKE simulations to generate rainfall–inundation sample pairs covering a representative range of storm magnitudes for model training and testing.

#### Hydrodynamic model setup and simulation

A coupled MIKE flood-modelling framework was established to generate physically consistent rainfall-inundation samples. MIKE URBAN was used to represent the urban drainage-network hydrodynamics, while MIKE 21 was used to simulate two-dimensional surface-flow and inundation processes. The numerical model was calibrated and checked against observed ponding depths from the 20 July 2021 storm event in Zhoukou City. The selected model parameters reported in the current manuscript included riverbed roughness values of 0.025–0.030 for the river component, dry and wet thresholds of 0.002 and 0.003 in MIKE 21, an eddy viscosity of 0.5, a Manning number of 32 for the surface model, an initial water depth of 0, an initial rainfall loss of 0.5, a decay coefficient of 0.9, and a pipe roughness of 0.015. The validated MIKE framework was then used to simulate multiple rainfall scenarios and generate the inundation-depth series required for deep-learning model development. Within this framework, MIKE URBAN was used to simulate rainfall-runoff generation and one-dimensional pipe-flow processes in the drainage system. The rainfall-runoff module calculated runoff generated from each contributing catchment, and these runoff volumes were supplied to the pipe-flow module as boundary inputs. The one-dimensional hydraulic routing in the drainage network was based on the Saint-Venant equations and solved numerically using the implicit Abbott-Ionescu six-point finite-difference scheme, allowing simulation of free-surface flow, pressurized flow, backflow, and overflow in branched urban drainage systems. MIKE 21 was used to simulate the two-dimensional surface inundation process in the urban area. Because the study area was large and the objective was to maintain computational efficiency while representing roads, buildings, and other urban structures at city scale, a structured grid was adopted. The governing equations were the depth-averaged two-dimensional Saint-Venant equations, including the continuity equation and momentum equations in the x and y directions. The variables included time, Cartesian coordinates, water depth, bed elevation, and the depth-averaged velocity components. The source term represented lateral inflow, and bed shear stress was calculated with Manning’s formulation.

#### Neural network model development and validation

A one-dimensional convolutional neural network (1D-CNN) was developed to learn the mapping between rainfall sequences and inundation-depth responses. The architecture reported in the manuscript consisted of an input layer, a first one-dimensional convolutional layer, a pooling layer, a second convolutional layer, a second pooling layer, a fully connected layer, and an output layer. Rectified linear unit (ReLU) activation was used as the nonlinear activation function. Batch normalization was applied to stabilize feature distributions during training, and dropout was used to reduce overfitting. The model was implemented as a lightweight surrogate intended for rapid prediction under emergency-response conditions. The rainfall series were organized as time-sequence inputs to the 1D-CNN. Based on the current manuscript, rainfall at the current time step and the five preceding time intervals was used to predict inundation depth at the target time step, and the prediction window was then advanced in a rolling manner along the storm process. The rainfall time interval used in the manuscript was 3 min, and the total rainfall duration for each designed event was 3 h. For the present STAR Methods draft, the model is described conservatively as predicting inundation-depth responses derived from MIKE simulations at designated target locations during the storm process. Rainfall-inundation sample pairs were generated from the validated MIKE simulations under the designed rainfall scenarios. Python scripts were used to batch-process the rainfall sequences and the corresponding inundation-depth outputs. According to the current manuscript, five inundation-risk control points were selected from each drainage area, and the resulting training dataset contained 432 samples. Before network training, rainfall inputs were normalized using min-max scaling to the range [0, 0.95], and the inverse transformation was applied during evaluation to restore the original physical units of inundation depth. The dataset was divided into two independent subsets, with 90% of the available rainfall-flooding samples used for training and the remaining 10% reserved as a held-out testing set. Hyperparameter tuning was conducted within the training subset using grid search combined with 5-fold cross-validation, and the final model performance was reported on the held-out test set. The current manuscript reports optimized epoch numbers and learning rates for the five rainfall sequences as follows: Serial 1, 230 epochs and 0.01; Serial 2, 200 epochs and 0.01; Serial 3, 150 epochs and 0.01; Serial 4, 110 epochs and 0.008; and Serial 5, 100 epochs and 0.008. A sample-size sensitivity analysis was also described, in which the 1D-CNN was trained using increasing fractions of the available dataset (for example, 20%, 40%, 60%, 80%, and 100%) to assess whether the 432 samples were sufficient. The test error reportedly decreased rapidly with sample size and then approached a plateau when the full dataset was used, indicating near-convergent performance. The numerical model was validated against observed ponding depths at 20 major waterlogging-prone locations for the 20 July 2021 event. The trained 1D-CNN was then evaluated against the numerical-model outputs and, for the same event, against observed or surveyed inundation depths at representative locations that were not included in training. In addition to process-based comparison between the MIKE and CNN hydrographs, feature-importance analysis was used to interpret the relative contribution of input factors to inundation-depth prediction. The current manuscript reports importance values for elevation, land-use type, drainage-network density, and rainfall return period, indicating that local topography and surface and drainage characteristics exerted stronger control on ponding depth than the return period within the designed 3-h scenario setting.

### Quantification and statistical analysis

#### Performance metrics

Model performance was quantified using six metrics: mean squared error (MSE), mean absolute error (MAE), coefficient of determination (R2), percent bias (PBIAS), Nash–Sutcliffe efficiency (NSE), and Kling–Gupta efficiency (KGE). MSE and MAE were used to evaluate the magnitude of prediction errors, R2 was used to indicate goodness of fit, PBIAS was used to diagnose overall bias, and NSE and KGE were used as integrated performance measures commonly adopted in hydrological-model evaluation. For MSE and MAE, smaller values indicate better agreement. For R2, values closer to 1 indicate better fit. For PBIAS, values close to 0 indicate low systematic bias, with positive values indicating overestimation and negative values indicating underestimation. For NSE and KGE, values closer to 1 indicate stronger predictive skill.

#### Data partitioning and cross-validation

The current manuscript states that 90% of the rainfall–flooding samples were used for model training and 10% were used for testing. Hyperparameter tuning was performed only within the training subset by combining grid search with 5-fold cross-validation. The final reported results therefore correspond to an independent held-out test set rather than to the cross-validation folds themselves.

#### Definition of n

For process-based prediction and metric calculation, n denotes the number of paired predicted and observed inundation-depth values included in a given evaluation. Depending on the analysis, this may correspond to the number of time steps in a hydrograph comparison, the number of target locations, or the number of test samples aggregated across locations and rainfall scenarios. In the current manuscript, PBIAS is reported at the location level for the 20 waterlogging-prone points, whereas NSE and KGE are reported as overall statistics across the 20 locations.

#### Reported results

For the 20 major waterlogging-prone sites analyzed for the 20 July 2021 event, the manuscript reports overall NSE and KGE values of 0.9671 and 0.9685, respectively. The average error between the CNN-predicted water depths and the measured water depths is reported as 5.74%, whereas the corresponding average error for the numerical model is reported as 6.97%. The manuscript also states that simulating a 3-h rainfall event required approximately 10 h in the numerical model but only about 8 s in the trained 1D-CNN surrogate.

#### Software

Numerical simulations were conducted with the MIKE modeling suite, and data processing and neural-network training were conducted in Python. The current manuscript should be checked before final submission to ensure that all Python libraries used for deep-learning implementation, data preprocessing, and plotting are explicitly listed, together with version numbers when available, in the key resources table and/or the main manuscript.
